# Searching for Innovative Functional Foods: Correlation Between Chemopreventive Potential and Bioactive Compounds Accumulation in Brassica Sprouts Grown Under Altered Gravity Conditions

**DOI:** 10.3390/ijms262311287

**Published:** 2025-11-22

**Authors:** Marta Markiewicz, Agnieszka Galanty, Paweł Zagrodzki, Agata Kołodziejczyk, Paweł Paśko

**Affiliations:** 1 Doctoral School of Medical and Health Sciences, Jagiellonian University Medical College, Łazarza 16, 31-530 Cracow, Poland; marta.markiewicz@doctoral.uj.edu.pl; 2 Department of Food Chemistry and Nutrition, Jagiellonian University Medical College, Medyczna 9, 30-688 Kraków, Poland; pawel.zagrodzki@uj.edu.pl; 3 Department of Pharmacognosy, Jagiellonian University Medical College, Medyczna 9, 30-688 Kraków, Poland; agnieszka.galanty@uj.edu.pl; 4 Faculty of Space Technologies, AGH University of Technology, 36 Czarnowiejska St., 30-054 Cracow, Poland; akolodziejczyk@agh.edu.pl

**Keywords:** functional food, thyroid cancer, polyphenols, sulfur compounds, Brassica sprouts, microgravity, darkness, nutraceutical, chemopreventive

## Abstract

The main aim of this study was to evaluate the effect of space-like environment on the chemopreventive activity of Brassica sprouts against thyroid cancer cells in vitro. For this purpose the sprouts of broccoli, kale, kohlrabi, and Brussels sprouts were cultivated in darkness and in microgravity for 5–7 days. Then, the sprouts’ extracts were examined for cytotoxic and antiproliferative activity against thyroid cancer and normal cells. The tested microgravity environment stimulated the cytotoxic activity of kohlrabi sprouts, causing approximately 50% reduction in thyroid cancer cells’ viability, while at the same time increasing the viability of normal thyroid cells. Broccoli sprouts showed the strongest antiproliferative activity against normal thyroid cells, with the best effect visible for darkness conditions, which may contribute to the reduction of thyroid hyperplasia. Microgravity and darkness significantly enhanced the antiproliferative activity of kale, especially in 7-day-old sprouts (inhibition approximately 90%). The tested conditions also increased the antiproliferative activity of kohlrabi sprouts, but in the case of Brussels sprouts the effect was unfavorable. The study showed that microgravity and darkness conditions may have significant influence on the chemopreventive role of Brassica sprouts against thyroid cancer cells in vitro, especially in the case of broccoli and kohlrabi sprouts.

## 1. Introduction

Brassica plants have been extensively studied for their chemopreventive activity [1] due to the presence of bioactive components such as sulfur compounds–glucosinolates and isothiocyanates [2,3]. Furthermore, despite some reports on their harmfulness to the thyroid, our recent review provided evidence that Brassica vegetables are not only safe for the thyroid (at adequate iodine supplementation) but may even inhibit the development of thyroid cancer [4]. Such properties allow us to include Brassica vegetables and their sprouts in the category of functional foods, understood as edible products that offer, in addition to basic nutrition, health-promoting properties. According to WHO data, the number of deaths from thyroid cancer more than doubled between 2000 and 2022, which proves the growing problem of this disease [5]. Because of this, it is important to propose new candidates for functional foods with potential chemopreventive activity toward thyroid cancer.

Why are Brassica sprouts promising in this context? Because of the very intense rate of plant growth and development at the sprouting stage, they are rich in bioactive compounds. Furthermore, they can be easily and quickly grown even at home, which facilitates their use in the daily diet. Some Brassica sprouts, such as broccoli and kale, have already been successfully studied for chemopreventive activity [6,7]; thus, we decided to study the hypothesis that unfavorable environmental conditions such as disturbed gravity or lack of sunlight can contribute to enhanced biological activity of the sprouts. Our previous results showed that these conditions induced stress in plants (observed as increased abscisic acid content) and caused a two-fold increase in polyphenolics’ synthesis in broccoli sprouts and a three-fold increase in sulfur compounds’ synthesis in kohlrabi sprouts. The antioxidant activity of kohlrabi sprouts also increased in microgravity and darkness conditions [8].

These results showed that sprouts grown in such unfavorable conditions may prove to be excellent candidates for functional foods and, thanks to their higher content of bioactive components, may also exert a stronger effect on inhibiting thyroid cancer development. To study this hypothesis, we used four selected Brassica species sprouts (broccoli, kale, Brussels sprouts, and kohlrabi), grown in disturbed gravity and darkness conditions, and tested their cytotoxic and antiproliferative activity toward human thyroid cancer cell lines (FTC-133, 8505C, TPC-1) and normal thyroid follicular epithelial cells (Nthy-ori 3-1). The chemometric approach was also used to show the potential relationship between the results obtained for the examined parameters.

## 2. Results and Discussion

The number of studies concerning the effect of disturbed gravity on the bioactivity of plants is small [8,9,10], but their results indicate that this may be a promising direction for obtaining functional food. Additionally, darkness conditions were previously reported as beneficial to the overall nutritional quality of Brassica sprouts [11]. Brassica sprouts are an important source of bioactive compounds, with sulfur representatives, namely glucosinolates (GLSs) and isothiocyanates (ITCs), typical metabolites for Brassica plants [12] and polyphenol–plant secondary metabolites, synthesized through the phenylpropanoid pathway [13]. Our previous results indicated that polyphenolic compounds dominated in broccoli, kale, and Brussels sprouts, while in kohlrabi sprouts sulfur compounds occurred in the greatest amount. We have also shown that microgravity combined with darkness significantly enhanced these bioactive compounds’ synthesis, especially in broccoli and kohlrabi sprouts. As far as bioactivity is concerned, darkness conditions during sprouting time increased the antioxidant activity of the tested Brassica sprouts, except kale. Microgravity, on the other hand, stimulated the antioxidant activity of kale, and in combination with darkness it also stimulated the antioxidant activity of kohlrabi [8]. These promising results led us to the question of whether the chemopreventive activity of Brassica sprouts would also be enhanced by microgravity and darkness.

### 2.1. Cytotoxic Activity Results

**Figure 1:**
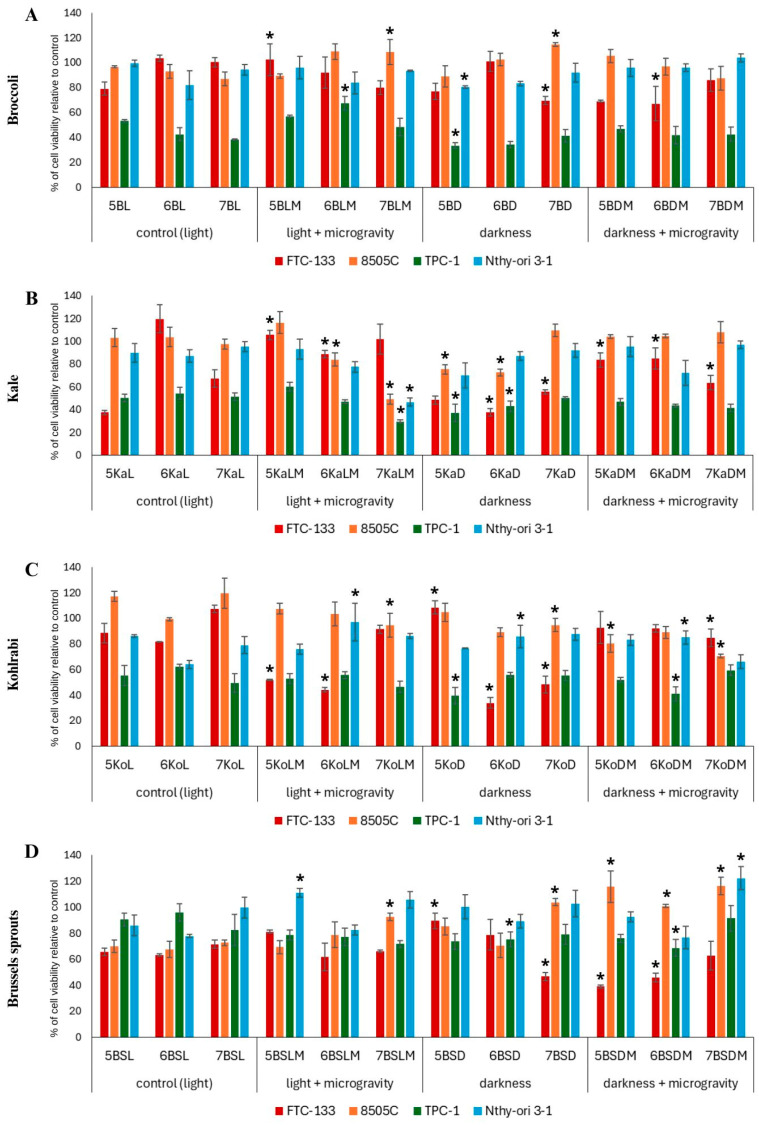
Viability of thyroid cancer (FTC-133, 8505C, and TPC-1) and normal thyroid (Nthy-ori 3-1) cells after 24 h incubation with the Brassica sprout extracts (broccoli (**A**), kale (**B**), kohlrabi (**C**) and Brussels sprouts (**D**)) used at a concentration of 300 μg/mL. Values are presented as the mean % ± SD (standard deviation) of three independent experiments of cell viability compared to the control cells (not treated with sprout extracts). Doxorubicin was used as a reference standard (IC_50_ 4.0, >40, 3.9, 27.2 μg/mL for FTC-133, 8505C, TPC-1, and Nthy-ori 3-1 cells, respectively). Significant differences between the cells treated with the extracts from the Brassica sprouts grown in microgravity and/or darkness conditions versus control sprouts (for each plant and harvest day separately) are marked with an upper asterisk * (*p* ≤ 0.05). Abbreviations of the sprouts’ samples: B—broccoli; Ka—kale; Ko—kohlrabi; BS—Brussels sprouts; L—standard light (control); D—darkness; LM—standard light and simulated microgravity; DM—darkness and simulated microgravity. The numbers placed before each abbreviation indicate harvest days (5, 6, and 7 days).

To perform the biological analysis of the examined sprouts, we used a specially designed panel of thyroid cancer cell lines, differing in their origin and metastatic potential. Thus, we used human cells representing follicular, papillary, and anaplastic thyroid cancers to better address the complex and heterogenous nature of the thyroid tumor, consisting usually of cells with distinct properties. Additionally, to evaluate the selectivity and safety of the extracts, we also included a human non-cancerous, normal thyroid epithelial cell line. Our results showed that depending on the type of thyroid cancer cells used, the effect of individual Brassica sprouts varied. In most cases, the observed effects were dose-dependent. In Figure 1 we present the results for the cytotoxic effect of the tested sprouts’ extracts in the highest concentration used (300 µg/mL), and in Tables S1–S4, we also present the results for concentrations of 50 and 100 µg/mL. To verify that the cytotoxic activity was not solely due to the presence of the DMSO solvent, its effect on cells was also examined and no impact on cell viability was noted.

#### 2.1.1. Cytotoxicity of Broccoli Sprouts

The results for the cytotoxic effect of broccoli sprouts at the highest tested dose are presented in Figure 1A, while those for the lower doses are in Table S1. In the case of broccoli sprouts, the extracts induced the most profound cytotoxic effect on TPC-1 cells, decreasing their viability even to 40%, compared to the control. The most active extract was 5 days-old broccoli sprouts cultivated in darkness (5BD), and it is worth noting that this result was significantly better than the control (5BL). Furthermore, the extracts 6BD and 7BD also demonstrated notable activity in the highest concentration of 300 µg/mL, but without significant differences compared to the corresponding controls. Microgravity and standard light conditions did not significantly affect the cytotoxic activity of broccoli sprouts. On the other hand, microgravity and darkness conditions caused a significant decrease in FTC-133 cells’ viability, especially on days 5 and 6 of harvesting, compared to the control (L) conditions. Interestingly, the viability of 8505C cells was unaffected, and in some cases even increased. It is worth noting that the viability of normal Nthy-ori 3-1 thyroid cells was not significantly affected by broccoli sprout extracts, as compared to the effect observed for cancer cells, which implies their selectivity. The cytotoxic activity of broccoli sprouts’ extracts, grown in standard light conditions, against thyroid cancer cells FTC-133 and 8505C has been examined previously only once [6], with FTC-133 cells being more susceptible (IC_50_ 35.2 µg/mL) than 8505C cells (IC_50_ 114.1 µg/mL) and without toxicity against Nthy-ori 3-1 cells, which is comparable to our experiment. However, the activity of broccoli sprouts against FTC-133 cells in our study is much lower than in the previous study [6], which may be due to the different methods of cultivating or preparing the sprouts for extraction. What is interesting is that the use of both darkness and microgravity caused a 2-fold increase in phenolic acid synthesis and a 13-fold increase in sulfur compound synthesis in broccoli sprouts in comparison to the control [8], which could be responsible for the high cytotoxic activity against FTC-133 cells. In our experiment, broccoli sprouts showed much greater activity against TPC-1 cells, which has not yet been examined in any other study; thus, this is the first report on their activity toward this cancer cell line.

#### 2.1.2. Cytotoxicity of Kale Sprouts

The results for the cytotoxic effect of kale sprouts (Ka) at the highest tested dose are presented in Figure 1B, while those for the lower doses are in Table S2. In the case of kale sprouts, the strongest cytotoxic effect was observed for the sprouts grown in complete darkness (KaD). Particularly pronounced effects were observed for 6KaD and 5KaD extracts at the concentration of 300 µg/mL, which was cytotoxic to all tested cells. A marked decrease in 8505C and TPC-1 cell viability was also observed for the 7KaLM extract at a concentration of 300 µg/mL. Similar to the effect noted for broccoli, TPC-1 cells were also the most sensitive to the kale sprouts’ extracts, while darkness (D) alone and darkness/microgravity (DM) conditions promoted cytotoxic effects. Interestingly, in some cases, cell viability increased, as was particularly observed for FTC-133 cells and the sprouts grown in microgravity and standard light (5KaLM, 7KaLM) and in microgravity and darkness (5KaDM). Unfortunately, the examined extracts also affected the viability of normal Nthy-ori 3-1 cells, although only moderately. Kale sprouts were previously tested for cytotoxic activity against FTC-133 and 8505C thyroid cancer cells [7]. Although this study focused on enriching the sprouts with selenium, the sprouts grown in control conditions reduced the viability of FTC-133 cells to approximately 70% and that of 8505C cells to 85%, which was a better result than for most of our extracts in control conditions. However, in the cited study, the extracts were used at concentrations of 500 µg/mL, almost twice as high as in our study, which may partially explain the difference. In our study, the strongest cytotoxic effect was observed for the sprouts grown in total darkness, with a decrease in viability of FTC-133 cells to approximately 40%, and to approximately 70% in 8505C cells. However, the most pronounced cytotoxic effect was observed in TPC-1 cells. Microgravity and darkness in our study significantly enhanced the cytotoxic activity of kale, especially in 7-day-old sprouts. It is worth noting, however, that the most pronounced cytotoxic effects were observed for the extract from 7-day-old sprouts grown in microgravity and standard light, although no selectivity over normal thyroid cells was observed. What is interesting is that one of the most active kale sprouts, 7KaLM, had a lower content of bioactive compounds compared to the control (7KaL) [8].

#### 2.1.3. Cytotoxicity of Kohlrabi Sprouts

The results for the cytotoxic effect of kohlrabi sprouts (Ko) at the highest tested dose are presented in Figure 1C, while those for the lower doses are in Table S3. In kohlrabi sprouts, a significant increase in cytotoxic activity (approximately 50% reduction in viability) was observed in TPC-1 cells treated with 5KoD and 6KoDM extracts, and in FTC-133 cells treated with 5KoLM and 6KoD extracts, as well as 6KoLM, at the highest concentration tested. Similarly, the sprouts grown in different gravity and light conditions also reduced the viability of 8505C cells, which was especially noted for KoDM sprouts. Interestingly, in some cases the toxic effect on viability of normal thyroid cells was diminished when microgravity was used in combination with darkness or standard light. What is also worth mentioning is that kale (described above) and kohlrabi sprouts exerted notable cytotoxic effects against 8505C cells, which is mostly an uncommon observation, because of the scarce reports on the cytotoxic influence of food compounds on 8505C cells and the known resistance of these cells to anticancer treatment [14,15]. The only previously studied Brassica plants which affected 8505C cells were broccoli and kale sprouts [6,7]. Other research describing cytotoxic activity on 8505C cells are focusing rather on compounds isolated from Brassica plants such as 3,3′-diindolylmethane [16].

#### 2.1.4. Cytotoxicity of the Sprouts of Brussels Sprouts

The results for the cytotoxic effect of Brussels sprouts at the highest tested dose are presented in Figure 1D, while those for the lower doses are in Table S4. In the case of Brussels sprouts (BS), darkness (D) and the combination of darkness and microgravity (DM) resulted in a significant increase in the cytotoxic activity of the extracts against FTC-133 cells compared to the control conditions (L). In contrast to the other examined sprouts, in the case of Brussels sprouts, TPC-1 cells were the least sensitive to the extracts, and in the case of the 6BSLM extract, their viability even increased. On the other hand, in the case of 8505C cells, the moderate cytotoxic effect observed for the control sprouts grown in standard light was further attenuated by the use of microgravity and darkness conditions. However, the viability of normal cells was not reduced, and in some cases even increased (e.g., 5- and 7-day-old sprouts grown in combined light and microgravity conditions) in comparison to the control. It is difficult to compare our results with those of other authors since the research on the direct relationship between Brussels sprouts and thyroid cancer is very limited, and focuses mainly on the impact on thyroid parameters such as TSH, T3, or T4 [17,18].

### 2.2. Antiproliferative Activity Results

Apart from the determination of the cytotoxic effects of the examined extracts, we also evaluated their impact on the proliferation rates of the thyroid cancer and normal cells, which is another aspect of chemoprevention. To do this, we used two subcytotoxic doses of the examined sprout extracts (50 and 100 µg/mL), and observed the effect after 24, 48, and 72 h. With the increase in the concentration of the tested extracts, the antiproliferative activity was more pronounced, with the most spectacular scores noted after a prolonged incubation of 72 h.

#### 2.2.1. Antiproliferative Activity of Broccoli Sprouts

The results for the antiproliferative activity of broccoli sprouts are presented in Table 1 (100 µg/mL) and Table S5 (50 µg/mL). In the case of TPC-1 cells, the strong antiproliferative effect observed for the control sprouts, grown in standard light, was reduced by almost two-fold after the use of microgravity. Similarly, the combination of darkness and microgravity also attenuated the antiproliferative effect, as compared not only to the sprouts grown in standard light, but also to those grown in the darkness alone. The proliferation rate of 8505C cells was strongly inhibited, up to almost 40%, after 72 h, and the effect was most pronounced in 6- and 7-day-old sprouts grown in light and microgravity. What should be underlined is that in all tested conditions a marked decrease in proliferation of 8505C cells was noted up to 51–68%, while the above-described viability of these cells was almost unaffected. Such an effect, observed at a subcytotoxic dose of 100 µg/mL, is of great importance, as this particular anaplastic thyroid cancer cell line is known for its resistance to conventional therapy [15]. In the case of FTC-133 cells, a strong antiproliferative impact of the examined extracts was noted, with the best result for the sprouts grown in darkness conditions. No significant effect of microgravity was observed, except in 5BLM and 6BLM sprouts, in which it attenuated the antiproliferative effect by almost two-fold. Broccoli sprouts also showed marked antiproliferative activity against normal thyroid Nthy-ori 3-1 cells, with the strongest effect for the sprouts grown in darkness conditions (28.2–35.5% of proliferation inhibition). The use of microgravity in this case did not significantly influence the antiproliferative effect of the extracts. As can be seen, the effects are not as clear here, with the most pronounced effect observed in normal cells, which may be a positive effect contributing to the reduction of thyroid hyperplasia. So far, the antiproliferative activity of broccoli sprouts has been confirmed against PC-3 (prostatic adenocarcinoma cells) [19], A549 (lung carcinoma cells), HepG2 (hepatocellular carcinoma cells), and Caco-2 cells [20], but to our knowledge, no such data exists for thyroid cancer cells, so the results are described here for the first time.

#### 2.2.2. Antiproliferative Activity of Kale Sprouts

The results for the antiproliferative activity of kale sprouts are presented in Table 2 (100 µg/mL) and Table S6 (50 µg/mL). FTC-133 cells were the most susceptible to the extracts among the cancer cell lines tested, especially after 72 h of incubation, with a decrease in proliferation up to about 20%. However, no significant impact of microgravity nor darkness conditions on the activity was noted. A clear effect of kale sprout extracts was also observed in TPC-1 cells, where the 7KaLM extract strongly inhibited the proliferation to 35.2% after just 24 h and to 9.8% after 72 h. However, this is the only case of microgravity having an impact on kale sprout extracts’ antiproliferative activity. Interestingly, a similar strong effect was noted for 7-day-old kale sprouts grown in the darkness alone, inhibiting cell proliferation to 9.89% after 72 h of incubation. In the case of 8505C cells, the effect of the extracts after 24 and 48 h was negligible, while after 72 h cells’ proliferation they were more profoundly inhibited by 6- and 7-day-old sprouts grown in light combined with microgravity, as compared to the light alone. Normal thyroid cells were also susceptible to the extracts, with the proliferation inhibited to approximately 30–40% (microgravity and darkness (LM, DM, D)) after 24 h and 50–60% in the control condition (KaL). To our knowledge, the results for the antiproliferative activity of kale sprouts are published herein for the first time ever.

#### 2.2.3. Antiproliferative Activity of Kohlrabi Sprouts

The results for the antiproliferative activity of kohlrabi sprouts are presented in Table 3 (100 µg/mL) and Table S7 (50 µg/mL). Kohlrabi sprouts’ extracts revealed moderate to strong antiproliferative activity toward the cell lines examined. In the case of FTC-133 and 8505C cells, 24 and 48 h of incubation did not result in significant antiproliferative activity, and the effect was visible only after 72 h. The 5-day-old sprouts grown in microgravity and/or darkness (KoLM, KoD, KoDM) were the most active against 8505C cells, while in the case of FTC-133 cells, 5–7-day-old extracts grown in microgravity (KoLM) and 5KoDM revealed the highest activity. TPC-1 cells were most susceptible to the tested extracts, with 5KoLM extract being the most active, inhibiting the proliferation to 13.7%, which was significant compared to the control (49.8%). Kohlrabi sprouts were also active against normal thyroid cells, inhibiting the proliferation to approximately 30% after 72 h, and microgravity and darkness conditions enhanced the antiproliferative activity compared to the control (KoL). To our knowledge, the results for the antiproliferative activity of kohlrabi sprouts are published herein for the first time ever.

#### 2.2.4. Antiproliferative Activity of Brussels Sprouts’ Sprouts

The results for the antiproliferative activity of Brussels sprouts are presented in Table 4 (100 µg/mL) and Table S8 (50 µg/mL). TPC-1 cells were the most susceptible to the extracts, but the observed inhibition of cell proliferation was attenuated by the combination of microgravity and light, but not the darkness, where the antiproliferative effect was sustained. A significant decrease in the proliferation of FTC-133 cells was also noted, but without the effect of microgravity. The overall antiproliferative effect of the extracts on 8505C cells was good, taking into account the above-mentioned resistance of the cells to therapy. Interestingly, microgravity enhanced the effect, but only in 7-day-old sprouts grown in the light combined with microgravity and 5-day-old sprouts grown in the darkness combined with microgravity. The proliferation of normal thyroid cells was also inhibited by Brussels sprout extracts, to about 30% after 72 h of incubation, but without a clear effect of microgravity or darkness. To our knowledge, the results for the antiproliferative activity of Brussels sprouts’ sprouts are published herein for the first time ever.

### 2.3. Chemometric Analysis

A statistically significant PCA model of four significant principal components (t1, t2, t3, and t4) was derived. Two first principal components complied with rule R1 of cross-validation: a component is significant when the percentage of total variation predicted by component Q^2^ > LIMIT. The two next principal components complied with rule R2: a component is significant when at least the square root of the original parameters follows QP^2^ > LIMIT where QP^2^ is Q^2^ for individual parameters (the LIMITS computed by SIMCA-P v.9 software were in the range 0.0505–0.0548). Table S9 shows the details of the PCA model. The parameters that had the highest absolute values of their loadings on t2 were as follows (parameter loading values are given in brackets, detailed sample abbreviations in chemometric analysis are presented in the Supplementary Materials): 72h 8505C CV50 (−0.30), 24 h 8505C CV100 (−0.33), and 72 h 8505C CV100 (−0.30). Those on t4 were as follows: TPC-1 MTT 300 (0.30), Nthy-ori 3-1 MTT 300 (0.30), 72 h FTC-133 CV50 (−0.30), 24 h 8505C CV50 (0.37), and FTC-133 MTT 300 (−0.41), respectively (only t2 and t4 were then included in the final superior PLS model). The PCA model explained 66.9% of the variance in the original response parameters (more details about the PCA model can be found in the Supplementary Materials).

**Figure 2:**
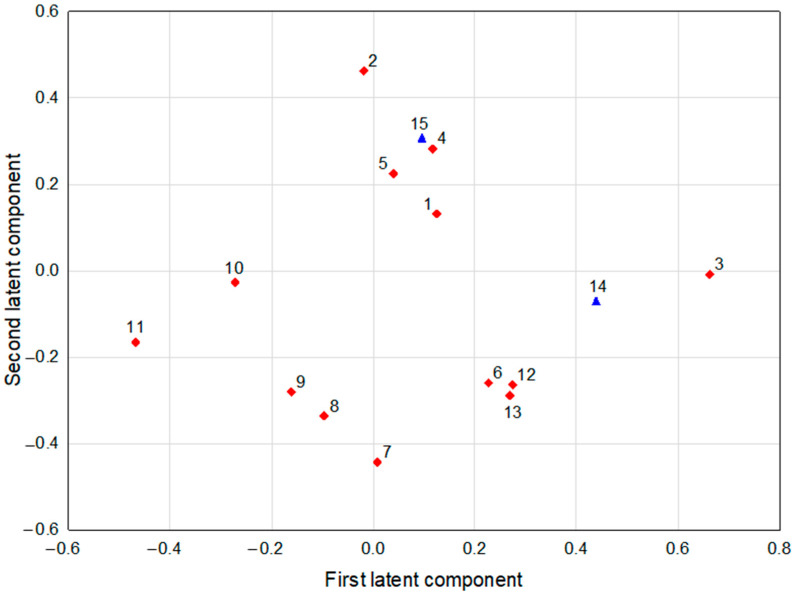
The loadings of the first and the second latent components in the PLS model (response parameters are depicted by blue triangles, predictor parameters by red diamonds; meaning of symbols: 1—protocatechuic acid, 2—caffeic acid, 3—chlorogenic acid, 4—isochlorogenic acid, 5—sinapic acid, 6—isoquercetin, 7—glucoiberin, 8—progoitrin, 9—glucoerucin, 10—sinigrin, 11—sulforaphane, 12—phenethyl isothiocyanate (phenethylNCS), 13—butyl isothiocyanate (butylNCS), 14—t2, 15—t4).

The PLS model fulfilling cross-validation criteria was constructed for predictor parameters and two combined parameters were obtained through PCA modeling (t2 and t4). A few other parameters (t1, t3, AllyloNCS) were not included in the model as they were considered noninformative (having very low loadings of both latent components, they were irrelevant to the problem of interrelationships under investigation and therefore were discarded from the model). The model had three significant components and explained R^2^X = 67.8% of variance in the predictive parameters and R^2^Y = 45.5% of variance in the response parameters, with eigenvalues of 2.92, 3.32, and 2.57, respectively. The cumulative fraction of total variation in response parameters that can be predicted by the extracted components was Q^2^cum = 16.1% (in PLS models, rules R1 and R2 are analogous to those in PCA models, but SIMCA applies a constant LIMIT for Q^2^ equal to 5%). The loadings for the first two latent components are shown in Figure 2.

The first latent component in this model had positive loadings predominantly for chlorogenic acid and t2 (which had high correlation coefficients among themselves) and negative loading for sulforaphane (Table 5). Therefore, chlorogenic acid correlated negatively with some of the CV results for the 8505C cell line (72 h 8505C CV50, 24 h 8505C CV100, and 72 h 8505C CV100), while sulforaphane had positive correlation coefficients with the above-mentioned parameters (Table 6). This suggests that the content of these two bioactive compounds may have a significant impact on the antiproliferative activity of the sprouts against 8505C cancer cells. The parameters loading mainly positively on the second latent component were caffeic acid and t4. Glucoiberin and progoitrin correlated negatively with this component (Table 5). Caffeic acid correlated positively with MTT results for TPC-1 and Nthy-ori 3-1 cells in the highest concentration used and CV results for 8505C cells (24 h 8505C CV50), which suggests that caffeic acid content may have a positive impact on the cytotoxic and antiproliferative activity of sprouts against TPC-1, 8505C, and Nthy-ori 3-1 cells. At the same time caffeic acid was negatively correlated with some of the results for FTC-133 cells (72 h FTC-133 CV50 and FTC-133 MTT 300), and also the correlations of glucoiberin and progoitrin with these parameters showed the opposite signs. This means that the influence of caffeic acid, progoitrin, and glucoiberin on the cytotoxic and antiproliferative activity of sprouts may differ depending on the cell lines used.

**Figure 3:**
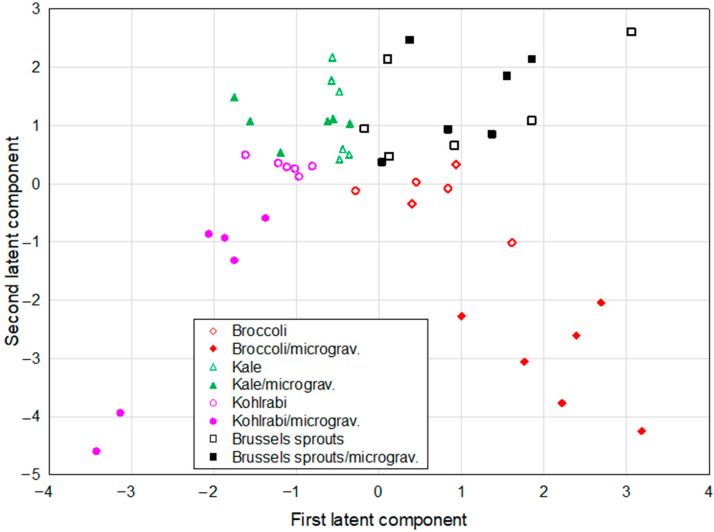
The projection of Brassica sprouts grown in different conditions defined by the first two latent components of the PLS model.

The projection of different species grown in different conditions (either in control conditions or in microgravity/darkness) was defined by the first two latent components of the PLS model and is shown in Figure 3. Analysis revealed two distinct clusters, each of them containing two different sprout species grown in microgravity conditions—broccoli and kohlrabi.

**Figure 4:**
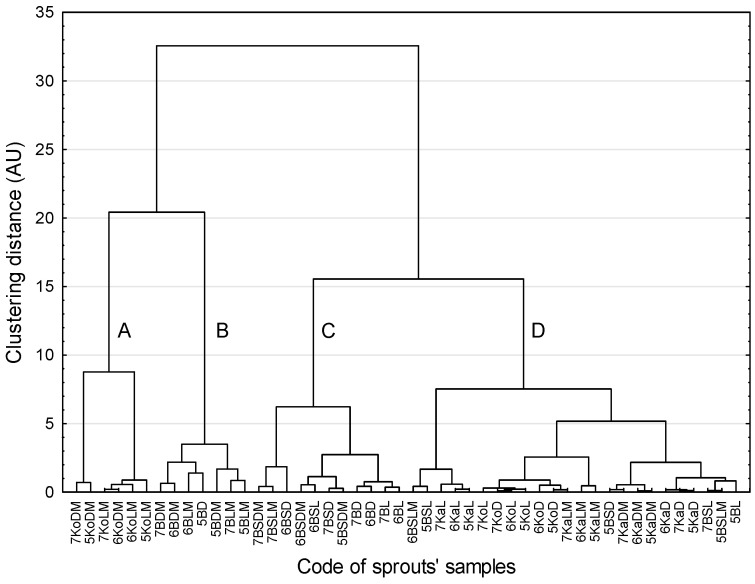
Dendrogram of similarity in sprout samples (method of grouping: Ward agglomeration procedure, function of the distance: Euclidean distance, Mojena’s rate: d = 10.18; subsequently identified clusters are marked with subsequent letters; further explanation in the text).

Essentially, the same clusters of the sprouts were confirmed by the CA method (Figure 4). The sprouts that were most distinct from the other sprouts were kohlrabi and broccoli. Clear similarities between the sprouts were revealed for the members of cluster A, i.e., kohlrabi sprouts grown in microgravity, and the members of cluster B, i.e., broccoli sprouts grown in microgravity, with only one wrongly classified member (5BD). The other two clusters (C and D) comprised sprouts of various species grown under different conditions.

Broccoli sprouts indeed differed in the studied parameters from the other species, especially in the context of the active compound content. For example, microgravity conditions caused the most prominent increase in the content of sulfur compounds in broccoli sprouts, which was the most visible change among all studied species [8]. Microgravity increased the total content of bioactive compounds in kohlrabi sprouts, which is reflected by the second cluster containing the samples of kohlrabi sprouts grown in microgravity. The analysis suggests that some phenolic acids (chlorogenic acid, caffeic acid) and glucosinolates (glucoiberin, progoitrin) played substantial but opposing roles in modulating cell responses. In particular, chlorogenic acid and sulforaphane appeared to act as antagonistic markers in relation to cell viability, while caffeic acid versus glucosinolates (glucoiberin, progoitrin) showed contrasting influence on cancer cell line responses; however, the correlations between these parameters do not imply causal or mechanistic relationships.

Based on the PLS model results it can be concluded that microgravity conditions can influence the bioactive compound accumulation and, because of that, indirectly the bioactivity of broccoli and kohlrabi sprouts. This suggests significant alterations due to microgravity, especially in broccoli and kohlrabi sprouts, which is consistent with the prior research highlighting environmental conditions altering plant biochemistry [21,22]. The content of sulfur compounds such as sulforaphane, progoitrin, and glucoiberin and polyphenolic compounds like chlorogenic acid and caffeic acid may have a significant impact on the antiproliferative and cytotoxic activity of the sprouts against thyroid cancer cells, and this influence largely depends on the cells used for the research.

## 3. Materials and Methods

### 3.1. Plant Material

Four different Brassica sprout species were used in this study: broccoli (*Brassica oleracea* var. *italica*, voucher specimen No.: 96713CIN0DS), kale (*Brassica oleracea* L., voucher specimen No.: 86912CIN0ES), both obtained from the TORAF sp. z o.o. company, Kujakowice Górne, Poland, kohlrabi (*Brassica oleracea* var. *gongylodes*, voucher specimen No.: PL014/32/83/NZ025A), obtained from the PlantiCo Hodowla i Nasiennictwo Ogrodnicze Zielonki Sp. z o.o. company, Zielonki, Poland, and Brussels sprouts (*Brassica oleracea* var. *Groningen*, voucher specimen No.: R47/F61), obtained from the W. Legutko Przedsiębiorstwo hodowlano-nasienne company, Jutrosin, Poland. The detailed procedure for growing sprouts was described previously [9]. Briefly, the seeds were first soaked in water for 30 min and transferred to specialized containers. These containers were rotated in a random positioning machine (RPM) throughout the cultivation period to simulate microgravity conditions. The sprouts were grown in standard light or complete darkness, at 22 ± 1 °C, for 5, 6, or 7 days. The growth conditions (light/darkness and temperature) were the same for control and experimental sprouts. Different cultivation conditions were denoted as follows: L—for standard light conditions, D—for darkness, and M—for microgravity conditions. Different species were denoted as follows: B—broccoli, Ka—kale, Ko—kohlrabi, and BS—Brussels sprouts. The numbers placed before each abbreviation indicate the respective harvest days (5, 6, or 7 days).

### 3.2. Microgravity Setup

To simulate microgravity conditions, the RPM (Astrotech, Cracow, Poland) was used, and the whole methodology has been described previously [9]. Because of the randomization of the rotation movement of two axes, the RPM simulates the behavior of biological specimens in free fall conditions. Microgravity simulation was achieved through the random deviation of the inner and outer ring movements, with an average rotational speed of 60 rpm for each ring.

### 3.3. Extraction

The whole extraction process has been described previously [9]. Briefly, sprouts were crushed in a mortar and extracted in a Soxhlet apparatus, using methanol as a solvent. The residues of the mentioned extracts were evaporated and dried to dry mass, and then 10 mg of the dry extracts was dissolved in 1 mL of DMSO.

### 3.4. Cell Cultures

Cytotoxic and antiproliferative activity was tested on human thyroid cancer cells—follicular thyroid carcinoma FTC-133, undifferentiated thyroid carcinoma 8505C, and papillary thyroid carcinoma TPC-1—and normal thyroid cells: thyroid follicular epithelial cells Nthy-ori 3-1. All cell lines were purchased in Merck (Darmstadt, Germany). The cells were grown under standard conditions, as mentioned previously [7]. The cells were seeded in 96-well plates for 24 h (1.5 × 10^4^ cells/well). The examined extracts were diluted in the culture media from freshly made stock solution in DMSO (10 mg/mL) to the working concentrations. The final concentration of DMSO in the wells did not exceed 0.1%. The culture medium was replaced with a fresh medium containing different concentrations of the extracts tested (50–300 μg/mL) and incubated for 24 h (cytotoxic activity) or 24, 48, and 72 h (antiproliferative activity). After that time, the cytotoxicity and antiproliferative activity were assessed.

### 3.5. Cytotoxic Activity

The viability of the cells was examined as described previously [9], using an MTT assay according to the manufacturer’s instructions. Each experiment was carried out in triplicate (three independent experiments). The absorbance was measured at 570 nm using a BioTek Synergy micro-plate reader (BioTek Instruments Inc., Winooski, VT, USA). Cell viability was expressed as percent of control, untreated cells. Doxorubicin was used as a reference standard. Additionally, the effect of DMSO on cell viability was tested.

### 3.6. Antiproliferative Activity

After 24, 48, and 72 h of incubation with the tested extracts, the cell number was determined using a crystal violet (CV) assay, as described previously [23]. Briefly, the cells were washed with PBS and washed with 3.7% formaldehyde. Then, CV solution was added for 10 min, followed by washing with PBS. Crystal violet was extracted from cells using 1.33% citric acid and 1.09% sodium citrate in a water/methanol (1:1) solution. The absorbance was measured at 570 nm using a BioTek Synergy micro-plate reader (BioTek Instruments Inc., Winooski, VT, USA). The proliferation rate was determined as a % of control, untreated cells.

### 3.7. Statistical Analysis

Statistical analysis was conducted using one-way ANOVA followed by a post hoc Tukey’s test. All experiments were performed in triplicate (three independent experiments), and the results were presented as the mean ± standard deviation (SD). Differences between groups were considered statistically significant at *p* values ≤ 0.05. A mixed hierarchical approach, including principal component analysis (PCA) and partial least squares (PLS) models, was used to reveal the correlation structure between the studied parameters and to increase the interpretability of the results (A, B). Firstly, a PCA model was constructed for a subset of all response parameters (results of MTT and CV tests on different cell lines in different cultivation conditions) to reduce the dimensionality of this subset of the data and create a significantly smaller number of new variables computed as principal components. The new variables were then used as response variables in a superior (hierarchical) PLS model in which remaining parameters, i.e., concentrations of polyphenols and sulfur compounds (data from our previous studies [8]), were used as predictor parameters. Before performing calculations, all variables were mean-centered and scaled to unit variance. We applied the explained-variance criterion: at least 50% of the variance in the parameters in the PCA model and at least 50% of the variance in the explanatory parameters in the PLS model had to be explained. It was assumed that parameters with the highest loadings (absolute value ≥ 0.3) in the PCA and PLS plots determine the axes (principal or latent components, respectively) of the new coordinate systems (in the PCA or PLS model) to the greatest extent. The parameters were considered negatively correlated if their weights within the PCA or PLS model showed the opposite signs. Otherwise, they were considered positively correlated. The association between two parameters was quantified by calculating correlation coefficients, i.e., the cosine of the corresponding angle (the angle determined by two lines connecting the origin with coordinates of both parameters on the PCA or PLS plot, respectively). The PLS approach was also applied to check whether the clusters of different plants grown in different conditions appear in its score plot. A cluster analysis (CA) approach was used to test whether clusters of plants that appeared in the PLS score plot would be confirmed by another multivariate statistical method. CA used the Ward agglomeration procedure as a method of grouping, and Euclidean distance as a function of the distance. The calculations of the PCA and PLS models were carried out with the package SIMCA-P v. 9 (Umetrics, Umeå, Sweden). The package STATISTICA v. 13.3. (TIBCO Software Inc., Palo Alto, CA, USA) was used for graphic representation of data [24,25].

## 4. Conclusions

The examined Brassica species sprouts’ extracts demonstrated varied effects on the tested thyroid cancer cell lines, ranging from strong to moderate, both by killing the cells and by limiting their growth. Thyroid cancer cells’ proliferation was inhibited at lower concentrations than those needed to achieve a decrease in cell viability. This double-track impact on thyroid cancer cells of the tested sprouts, combined with their antioxidant activity, demonstrated in our previous study, proved that Brassica sprouts of the examined species perfectly fit the idea of chemoprevention, meeting the requirements for candidates for functional foods. The observed chemopreventive potential of the examined sprouts deserves further studies, directed at the mechanism of action or the impact on animal organisms. The observed antiproliferative effects of most of the tested extracts on normal thyroid Nthy-ori 3-1 cells may be considered as positive, contributing to the reduction of thyroid hyperplasia. However, this effect needs to be examined in depth in future studies.

We have proven that sprouts’ bioactivity can be moderated by the conditions of microgravity and darkness, related to the change in the active compounds’ composition of Brassica sprouts. This influence of the microgravity environment was especially visible in the case of broccoli and kohlrabi sprouts, which was demonstrated and confirmed with the use of advanced chemometric analysis. The marked decrease in proliferation of 8505C cells in all tested conditions is of great importance, as this particular cell line is known for its resistance to conventional therapy. Our results also constitute evidence that microgravity conditions can influence the bioactive compound accumulation and indirectly the bioactivity of Brassica sprouts.

## Figures and Tables

**Table 1:** The antiproliferative activity of broccoli sprouts (100 µg/mL) against thyroid cancer and normal cells.

	5BL	6BL	7BL	5BLM	6BLM	7BLM	5BD	6BD	7BD	5BDM	6BDM	7BDM
FTC-133												
24 h	113.51 ± 1.29	79.65 ± 6.21	82.81 ± 7.11	90.06 ± 6.3	94.44 ± 7.86	80.27 ± 3.38	83.83 ± 3.53 *	68.64 ± 8.56	71.70 ± 9.65	67.72 ± 11.10 *	94.75 ± 14.81	72.82 ± 6.63
48 h	98.48 ± 6.02	61.19 ± 8.82	73.88 ± 6.24	71.54 ± 5.06 *	75.28 ± 9.12	88.94 ± 8.96	86.67 ± 11.00	70.24 ± 5.06	72.36 ± 7.35	94.20 ± 4.35	86.61 ± 5.05 *	91.46 ± 8.62
72 h	40.84 ± 0.48	31.62 ± 4.10	35.20 ± 5.48	68.13 ± 7.13 *	59.43 ± 4.56 *	35.34 ± 3.73	27.20 ± 3.50 *	25.31 ± 2.16	28.82 ± 5.22	32.61 ± 4.78	39.57 ± 6.76	26.77 ± 1.76
8505C												
24 h	88.93 ± 2.22	91.28 ± 6.35	84.35 ± 3.42	90.10 ± 7.82	89.25 ± 4.52	83.97 ± 9.73	80.40 ± 7.10	92.66 ± 7.16	84.99 ± 3.79	86.91 ± 6.44	93.41 ± 7.23	89.73 ± 1.13
48 h	112.72 ± 4.09	96.98 ± 12.20	78.23 ± 4.00	84.14 ± 8.92 *	81.31 ± 2.70	87.99 ± 6.52	82.47 ± 3.06 *	87.48 ± 6.48	83.75 ± 6.48	87.09 ± 5.39 *	86.71 ± 7.58	105.97 ± 5.34 *
72 h	64.35 ± 9.62	68.43 ± 7.07	51.55 ± 4.00	61.03 ± 9.89	42.60 ± 2.24 *	43.96 ± 6.26	51.96 ± 1.64	54.61 ± 4.71	52.11 ± 2.95	61.18 ± 3.12	58.69 ± 6.50	66.92 ± 9.06
TPC-1												
24 h	91.68 ± 1.84	75.02 ± 10.00	62.53 ± 2.27	92.79 ± 10.30	84.50 ± 8.60	77.35 ± 9.39	63.79 ± 3.07 *	65.40 ± 8.12	74.08 ± 7.78	76.29 ± 2.14	85.77 ± 1.06	91.65 ± 8.45 *
48 h	62.16 ± 6.86	47.30 ± 2.59	41.74 ± 2.33	92.93 ± 6.54 *	85.60 ± 6.02 *	71.80 ± 2.03 *	44.76 ± 7.34	32.26 ± 5.02	44.93 ± 4.78	46.01 ± 7.31	53.47 ± 11.36	61.71 ± 6.56 *
72 h	33.88 ± 3.41	32.99 ± 2.3	26.13 ± 2.30	54.55 ± 6.65 *	53.40 ± 3.58 *	53.77 ± 2.27 *	12.82 ± 0.31 *	36.62 ± 5.17	41.97 ± 3.71 *	41.60 ± 8.75	50.85 ± 1.97 *	42.36 ± 4.26 *
Nthy-ori 3-1												
24 h	55.09 ± 8.29	46.99 ± 1.82	48.11 ± 2.19	40.46 ± 6.01	34.45 ± 5.71	37.04 ± 6.54	35.49 ± 7.88 *	26.21 ± 2.24 *	28.21 ± 5.56 *	28.88 ± 3.69 *	32.96 ± 7.52	31.85 ± 5.40
48 h	31.36 ± 0.50	35.71 ± 4.70	34.22 ± 1.78	35.87 ± 6.42	36.81 ± 5.76	33.83 ± 6.12	33.20 ± 5.66	33.20 ± 4.51	34.69 ± 3.37	34.93 ± 3.53	33.83 ± 4.69	38.21 ± 2.83
72 h	29.83 ± 3.77	29.23 ± 4.11	30.53 ± 0.80	29.67 ± 5.42	25.67 ± 3.24	27.40 ± 1.17	24.70 ± 1.21	23.62 ± 1.75	24.49 ± 1.55	25.78 ± 0.67	25.00 ± 3.75	24.81 ± 1.80

The results are presented as the mean % of cell proliferation inhibition ± SD (standard deviation) of three independent experiments. Significant differences between the cells treated with the extracts from the Brassica sprouts grown in microgravity and/or darkness conditions versus control sprouts are marked with an upper asterisk * (*p* ≤ 0.05). Abbreviations of the sprouts’ samples: B—broccoli; L—standard light (control); D—darkness; LM—standard light and simulated microgravity; DM—darkness and simulated microgravity. The numbers placed before each abbreviation indicate harvest days (5, 6, and 7 days).

**Table 2:** The antiproliferative activity of kale sprouts (100 µg/mL) against thyroid cells.

	5KaL	6KaL	7KaL	5KaLM	6KaLM	7KaLM	5KaD	6KaD	7KaD	5KaDM	6KaDM	7KaDM
FTC-133												
24 h	85.21 ± 6.71	83.68 ± 7.14	73.53 ± 8.29	76.08 ± 9.66	80.37 ± 1.57	74.35 ± 0.81	93.32 ± 1.73	83.22 ± 9.79	73.43 ± 14.18	77.21 ± 11.30	83.32 ± 9.40	67.31 ± 10.20
48 h	60.65 ± 5.30	62.28 ± 6.46	51.27 ± 5.60	62.49 ± 4.61	60.65 ± 6.55	56.64 ± 2.68	66.99 ± 8.40	54.96 ± 5.29	53.93 ± 3.61	62.66 ± 7.45	56.26 ± 3.36	56.86 ± 1.88
72 h	29.29 ± 6.86	33.22 ± 3.48	24.59 ± 0.66	26.66 ± 4.83	24.021 ± 3.16	22.61 ± 6.14	26.96 ± 3.59	18.56 ± 2.91 *	19.05 ± 3.39	24.51 ± 6.52	22.94 ± 5.26	19.53 ± 0.40
8505C												
24 h	112.07 ± 3.20	104.93 ± 4.73	103.81 ± 13.80	115.48 ± 4.07	101.73 ± 0.32	98.10 ± 7.98	99.59 ± 9.69	95.22 ± 0.18	100.23 ± 2.44	97.57 ± 13.60	113.56 ± 0.90	104.77 ± 4.30
48 h	117.53 ± 0.54	107.77 ± 2.35	109.31 ± 5.89	109.06 ± 8.83	101.86 ± 6.36	91.20 ± 2.35	97.62 ± 3.86 *	101.73 ± 8.09	104.30 ± 4.88	102.63 ± 4.05	106.74 ± 13.70	124.66 ± 1.91
72 h	85.73 ± 8.09	88.14 ± 4.31	77.72 ± 5.36	71.15 ± 5.49	67.07 ± 2.56 *	54.46 ± 4.53 *	69.11 ± 9.05	78.40 ± 4.90	66.77 ± 4.28	79.23 ± 7.72	79.31 ± 6.17	79.53 ± 7.16
TPC-1												
24 h	69.61 ± 1.95	67.73 ± 5.41	62.93 ± 2.62	58.32 ± 3.48	53.97 ± 4.21 *	35.17 ± 4.68 *	71.27 ± 2.23	70.61 ± 4.65	55.41 ± 1.56	73.48 ± 3.94	70.14 ± 7.09	76.22 ± 8.05 *
48 h	38.32 ± 4.90	43.14 ± 1.40	29.89 ± 4.40	28.05 ± 0.00	29.98 ± 7.93	22.72 ± 1.83	56.14 ± 8.97 *	40.27 ± 7.36	21.66 ± 4.65	39.30 ± 1.62	36.06 ± 3.35	36.20 ± 2.29
72 h	32.59 ± 3.23	40.14 ± 2.90	43.87 ± 5.97	47.22 ± 5.16 *	35.55 ± 4.47	9.83 ± 4.33 *	43.25 ± 5.01	34.14 ± 4.41	9.89 ± 0.85 *	35.71 ± 3.03	37.52 ± 5.86	38.78 ± 7.87
Nthy-ori 3-1												
24 h	61.69 ± 7.56	59.24 ± 1.89	51.67 ± 4.83	44.99 ± 6.65 *	42.32 ± 4.65 *	38.45 ± 3.66 *	32.29 ± 4.60 *	38.31 ± 4.16 *	43.80 ± 2.82	40.91 ± 2.69 *	36.90 ± 3.07 *	39.57 ± 2.94
48 h	53.25 ± 5.67	50.90 ± 9.05	42.52 ± 8.77	37.51 ± 10.10	41.89 ± 7.69	38.06 ± 3.95	36.34 ± 4.80	40.02 ± 7.11	33.99 ± 4.67	31.87 ± 1.06 *	32.58 ± 2.59	34.53 ± 1.63
72 h	47.36 ± 3.82	41.32 ± 4.84	37.86 ± 5.88	38.35 ± 5.63	32.90 ± 1.60	29.02 ± 3.91	27.18 ± 3.98 *	29.56 ± 5.15	25.94 ± 2.94	27.02 ± 6.03 *	27.62 ± 4.20 *	27.94 ± 5.66

The results are presented as the mean % of cell proliferation inhibition ± SD (standard deviation) of three independent experiments. Significant differences between the cells treated with the extracts from the Brassica sprouts grown in microgravity and/or darkness conditions versus control sprouts are marked with an upper asterisk * (*p* ≤ 0.05). Abbreviations of the sprouts’ samples: Ka—kale; L—standard light (control); D—darkness; LM—standard light and simulated microgravity; DM—darkness and simulated microgravity. The numbers placed before each abbreviation indicate harvest days (5, 6, and 7 days).

**Table 3:** The antiproliferative activity of kohlrabi sprouts (100 µg/mL) against thyroid cells.

	5KoL	6KoL	7KoL	5KoLM	6KoLM	7KoLM	5KoD	6KoD	7KoD	5KoDM	6KoDM	7KoDM
FTC-133												
24 h	133.10 ± 10.82	117.03 ± 0.22	101.58 ± 9.14	93.73 ± 5.05 *	98.06 ± 11.47 *	101.17 ± 9.22	112.29 ± 6.06 *	108.92 ± 9.09	106.48 ± 5.62	91.79 ± 4.01 *	106.02 ± 1.08	102.19 ± 3.53
48 h	110.81 ± 11.84	113.88 ± 11.20	94.91 ± 8.39	84.39 ± 0.92 *	104.66 ± 6.69	85.80 ± 2.22	84.28 ± 9.86 *	111.49 ± 10.80	108.83 ± 2.33	100.38 ± 8.54	98.54 ± 12.73	110.41 ± 4.69
72 h	80.53 ± 5.02	76.72 ± 7.78	85.93 ± 8.53	51.55 ± 5.47 *	54.79 ± 9.48 *	56.02 ± 8.71 *	66.20 ± 3.99	69.61 ± 4.48	67.73 ± 3.58 *	44.70 ± 0.23 *	61.62 ± 4.59	67.80 ± 0.40 *
8505C												
24 h	119.00 ± 5.43	105.25 ± 9.47	106.31 ± 9.33	103.11 ± 5.12	112.71 ± 5.47	102.15 ± 9.94	103.97 ± 6.97	97.68 ± 7.68	102.79 ± 6.66	109.40 ± 3.32	111.96 ± 6.33	99.01 ± 4.30
48 h	125.43 ± 0.82	105.84 ± 4.05	104.62 ± 3.54	91.33 ± 1.77 *	108.16 ± 4.79	111.37 ± 7.63	113.55 ± 6.63	110.60 ± 0.39	103.92 ± 4.79	101.35 ± 6.06 *	108.28 ± 0.00	96.53 ± 2.00 *
72 h	101.81 ± 7.40	90.11 ± 3.21	83.31 ± 6.00	71.75 ± 1.76 *	77.57 ± 4.89	97.09 ± 17.50	79.98 ± 6.30 *	82.85 ± 3.51	73.34 ± 5.59	75.11 ± 5.29 *	92.22 ± 9.91	95.62 ± 9.31
TPC-1												
24 h	80.63 ± 4.29	63.13 ± 10.30	71.21 ± 2.41	47.29 ± 1.22 *	79.69 ± 6.32	74.08 ± 8.57	85.44 ± 5.53	84.30 ± 2.34 *	88.71 ± 8.52	82.77 ± 9.08	85.24 ± 8.72 *	88.44 ± 8.16
48 h	66.57 ± 0.67	42.08 ± 1.77	43.75 ± 5.48	22.059 ± 4.88 *	62.16 ± 8.25 *	57.43 ± 5.64 *	55.19 ± 4.94	55.82 ± 3.22 *	59.98 ± 5.10 *	55.48 ± 2.56	50.40 ± 2.83	56.25 ± 3.35
72 h	49.86 ± 0.32	33.91 ± 4.69	37.83 ± 9.79	13.73 ± 0.97 *	47.82 ± 3.94	51.51 ± 10.12	65.94 ± 4.23	50.06 ± 3.49	49.42 ± 4.36	49.24 ± 7.94	52.73 ± 6.77 *	62.22 ± 3.24 *
Nthy-ori 3-1												
24 h	76.39 ± 6.61	68.00 ± 4.59	52.41 ± 4.68	45.73 ± 6.13 *	51.60 ± 6.72 *	43.13 ± 3.21	36.75 ± 0.94 *	44.84 ± 5.39 *	49.67 ± 5.76	41.50 ± 3.76 *	40.53 ± 1.78 *	36.97 ± 2.52 *
48 h	64.53 ± 6.88	60.77 ± 1.76	47.53 ± 4.76	39.47 ± 7.64 *	41.50 ± 5.82 *	39.62 ± 3.30	39.94 ± 2.49 *	45.26 ± 2.18 *	51.53 ± 3.53	44.09 ± 2.94 *	43.46 ± 2.01 *	48.94 ± 7.09
72 h	51.67 ± 7.96	44.88 ± 6.85	37.22 ± 5.37	30.64 ± 3.14 *	31.55 ± 0.97	32.96 ± 2.46	28.37 ± 5.57 *	29.83 ± 2.35 *	36.73 ± 6.17	28.16 ± 2.97 *	30.20 ± 5.70 *	29.93 ± 4.86

The results are presented as the mean % of cell proliferation inhibition ± SD (standard deviation) of three independent experiments. Significant differences between the cells treated with the extracts from the Brassica sprouts grown in microgravity and/or darkness conditions versus control sprouts are marked with an upper asterisk * (*p* ≤ 0.05). Abbreviations of the sprouts’ samples: Ko—kohlrabi; L—standard light (control); D—darkness; LM—standard light and simulated microgravity; DM—darkness and simulated microgravity. The numbers placed before each abbreviation indicate harvest days (5, 6, and 7 days).

**Table 4:** The antiproliferative activity of Brussels sprouts (100 µg/mL) against thyroid cells.

	5BSL	6BSL	7BSL	5BSLM	6BSLM	7BSLM	5BSD	6BSD	7BSD	5BSDM	6BSDM	7BSDM
FTC-133												
24 h	131.57 ± 13.76	83.63 ± 3.68	79.96 ± 6.43	93.63 ± 13.10 *	77.82 ± 3.37	83.63 ± 7.78	96.58 ± 12.80 *	88.83 ± 9.46	79.35 ± 12.90	80.88 ± 6.46 *	81.39 ± 4.86	83.02 ± 10.20
48 h	112.52 ± 8.74	71.54 ± 5.52	77.15 ± 1.95	95.45 ± 1.84 *	78.78 ± 0.81	92.44 ± 9.08 *	78.86 ± 11.00 *	77.72 ± 2.07	79.27 ± 8.85	99.84 ± 9.16	74.47 ± 7.82	110.08 ± 2.75 *
72 h	63.12 ± 8.43	32.94 ± 5.55	29.29 ± 3.54	65.69 ± 9.09	27.95 ± 6.12	48.28 ± 9.54	53.25 ± 4.64	78.59 ± 12.70 *	30.58 ± 5.46	62.09 ± 3.11	47.99 ± 6.91	49.79 ± 3.14 *
8505C												
24 h	95.81 ± 10.60	93.73 ± 8.37	86.48 ± 5.69	82.11 ± 8.96	86.59 ± 6.74	86.69 ± 2.42	86.27 ± 8.97	90.64 ± 6.79	82.00 ± 5.69	85.63 ± 5.62	79.65 ± 5.43	90.53 ± 7.82
48 h	116.96 ± 9.54	100.71 ± 8.69	78.61 ± 5.45	83.37 ± 3.32 *	79.77 ± 8.72 *	103.79 ± 3.58 *	86.45 ± 7.27 *	75.92 ± 3.68 *	78.74 ± 6.27	78.23 ± 1.02 *	74.63 ± 11.40 *	95.18 ± 9.73
72 h	68.66 ± 7.65	71.37 ± 0.45	63.56 ± 3.68	66.09 ± 5.51	60.61 ± 11.70	43.73 ± 1.92 *	56.99 ± 2.40	52.23 ± 1.76 *	60.65 ± 4.16	49.92 ± 9.11 *	64.12 ± 7.71	68.73 ± 6.61
TPC-1												
24 h	92.52 ± 6.31	71.14 ± 1.84	67.40 ± 2.01	90.71 ± 8.37	64.73 ± 11.62	92.85 ± 3.95 *	86.17 ± 3.68	90.98 ± 6.86 *	74.35 ± 3.68	99.93 ± 1.98	101.20 ± 7.81 *	99.20 ± 11.34 *
48 h	65.98 ± 5.26	58.86 ± 4.76	43.98 ± 6.43	98.06 ± 7.63 *	41.74 ± 3.19	67.01 ± 9.08 *	76.07 ± 3.86	84.22 ± 9.12 *	48.51 ± 7.06	92.76 ± 7.85 *	84.56 ± 7.87 *	93.45 ± 3.15 *
72 h	36.81 ± 5.36	20.61 ± 4.56	21.26 ± 5.27	59.96 ± 7.06 *	21.52 ± 4.66	45.97 ± 8.62 *	53.85 ± 3.71	59.36 ± 7.97 *	35.62 ± 2.79	52.04 ± 3.18	58.57 ± 4.91 *	55.51 ± 2.27 *
Nthy-ori 3-1												
24 h	51.52 ± 4.31	45.29 ± 1.79	39.64 ± 3.28	44.25 ± 1.87	42.02 ± 6.63	43.95 ± 7.02	46.92 ± 8.59	44.10 ± 8.25	34.45 ± 3.79	43.88 ± 3.58	37.56 ± 9.86	36.82 ± 4.48
48 h	44.17 ± 4.98	46.12 ± 6.10	43.46 ± 2.31	41.35 ± 3.46	40.33 ± 5.73	41.93 ± 1.83	40.17 ± 6.54	38.21 ± 3.64	35.32 ± 6.20	35.87 ± 3.95	36.49 ± 1.11	43.93 ± 3.73
72 h	41.69 ± 3.64	36.14 ± 3.92	34.95 ± 7.01	34.25 ± 7.36	29.61 ± 0.97	30.42 ± 2.82	33.71 ± 3.33	29.83 ± 8.36	25.94 ± 2.30	26.27 ± 6.40 *	27.40 ± 3.05	27.56 ± 3.65

The results are presented as the mean % of cell proliferation inhibition ± SD (standard deviation) of three independent experiments. Significant differences between the cells treated with the extracts from the Brassica sprouts grown in microgravity and/or darkness conditions versus control sprouts are marked with an upper asterisk * (*p* ≤ 0.05). Abbreviations of the sprouts’ samples: BS—Brussels sprouts; L—standard light (control); D—darkness; LM—standard light and simulated microgravity; DM—darkness and simulated microgravity. The numbers placed before each abbreviation indicate harvest days (5, 6, and 7 days).

**Table 5:** Correlation coefficients for the pairs of parameters based on the PLS model.

Pairs of Correlated Parameters		Correlation Coefficient
First latent component		
chlorogenic acid	t2	0.990
sulforaphane	t2	−0.879
chlorogenic acid	sulforaphane	−0.938
Second latent component		
glucoiberin	progoitrin	0.957
caffeic acid	t4	0.941
glucoiberin	t4	−0.948
caffeic acid	progoitrin	−0.951
progoitrin	t4	−1.000
caffeic acid	glucoiberin	−1.000

**Table 6:** Correlations between predictive parameters and original response parameters, implied by correlations revealed in the PLS model (calculated as products of correlation coefficients between the relevant prediction parameters and t2 and the loadings of the response parameters most strongly influencing t2, and analogously for parameters related to t4).

**First Latent Component; Correlations Implied with Original Parameters**					
**Parameter**	**72 h 8505C CV50**	**24 h 8505C CV100**	**72 h 8505C CV100**		
chlorogenic acid	−0.297	−0.327	−0.297		
sulforaphane	0.261	0.287	0.261		
**Second Latent Component; Correlations Implied with Original Parameters**					
**Parameter**	**TPC-1 MTT 300**	**Nthy-ori 3-1 MTT 300**	**24 h 8505C CV50**	**72 h FTC-133 CV50**	**FTC-133 MTT 300**
caffeic acid	0.287	0.287	0.354	−0.287	−0.392
glucoiberin	−0.284	−0.284	−0.351	0.284	0.389
progoitrin	−0.300	−0.300	−0.370	0.300	0.410

Sample abbreviations: 72 h 8505C CV50—antiproliferative assay, concentration of 50 µg/mL, 72 h of incubation, 8505C cells; 24 h 8505C CV100—antiproliferative assay, concentration of 100 µg/mL, 24 h of incubation, 8505C cells; 72 h 8505C CV100—antiproliferative assay, concentration of 100 µg/mL, 72 h of incubation, 8505C cells; TPC-1 MTT 300—cytotoxic assay, concentration of 300 µg/mL, TPC-1 cells; Nthy-ori 3-1 MTT 300—cytotoxic assay, concentration of 300 µg/mL, Nthy-ori 3-1 cells; 24 h 8505C CV50—antiproliferative assay, concentration of 50 µg/mL, 24 h of incubation, 8505C cells; 72 h FTC-133 CV50—antiproliferative assay, concentration of 50 µg/mL, 72 h of incubation, FTC-133 cells; FTC-133 MTT 300—cytotoxic assay, concentration of 300 µg/mL, FTC-133 cells.

## Data Availability

The original contributions presented in this study are included in the article/Supplementary Materials. Further inquiries can be directed to the corresponding author(s).

## Supplementary Materials

The supporting information can be downloaded at https://www.mdpi.com/article/10.3390/ijms262311287/s1.
