# Supplementary material for: Searching for Innovative Functional Foods: Correlation Between Chemopreventive Potential and Bioactive Compounds Accumulation in Brassica Sprouts Grown Under Altered Gravity Conditions
Source: Int J Mol Sci. 2025 Nov 22;26(23):11287. doi: 10.3390/ijms262311287 (PMC12692239; doi:10.3390/ijms262311287)
Supplement: Supplementary file 1 [file ijms-26-11287-s001.zip › ijms-3931434-supplementary.pdf]

Table S1. Viability of thyroid cancer (FTC-133, 8505C and TPC-1) and thyroid normal (Nthy-ori 3-1) cells after 24 h incubation with broccoli sprouts' extract.

[μg/mL]	5BL	6BL	7BL	5BLM	6BLM	7BLM	5BD	6BD	7BD	5BDM	6BDM	7BDM
						FTC-133						
50	104.78 ± 4.61	109.43 ± 11.95	113.4 ± 6.88	98.14 ± 8.79	102.64 ± 8.62	87.05 ± 1.84	86.29 ± 9.66	109.47 ± 8.89	104.40 ± 15.22	73.05 ± 7.78*	81.14 ± 9.49	92.27 ± 5.62
100	122.39 ± 5.33	117.36 ± 5.17	91.7 ± 5.23	126.85 ± 2.42	107.52 ± 18.03	102.41 ± 13.84	106.00 ± 10.75	117.82 ± 0.97	76.94 ± 4.03	74.66 ± 5.49*	79.54 ± 7.22*	89.30 ± 7.31
						8505C						
50	80.66 ± 3.18	88.01 ± 4.18	86.3 ± 5.52	97.17 ± 1.49*	94.06 ± 1.87	91.53 ± 2.5	100.76 ± 2.78*	104.77 ± 7.92*	110.24 ± 1.99	119.4 ± 1.53*	97.49 ± 5.64	92.26 ± 6.13*
100	91.77 ± 2.48	90.3 ± 6.65	120.38 ± 5.2	97.58 ± 7.87	104.03 ± 5.33	102.81 ± 4.96	105.5 ± 9.55	110.32 ± 6.42*	111.14 ± 9.99	110.08 ± 3.31*	99.13 ± 5.24	82.62 ± 3.61*
						TPC-1						
50	101.78 ± 6.21	93.81 ± 2.91	86.55 ± 7.1	86.31 ± 4.89	98.66 ± 4.16	77.14 ± 11.69	92.79 ± 5.47	87.87 ± 3.38	81.76 ± 9.36	89.43 ± 3.52*	89.25 ± 6.42	91.65 ± 2.48
100	94.05 ± 2.54	80.08 ± 9.89	73.79 ± 8.93	86.43 ± 4.64	95.48 ± 9.04	82.96 ± 4.91	77.98 ± 8.09	77.56 ± 2.08	69.11 ± 7.48	82.3 ± 5.06	82.06 ± 7.99	70.07 ± 7.96
						Nthy-ori 3-1						
50	81.78 ± 3.78	107.88 ± 5.1	97.77 ± 4.17	96.65 ± 10.94	91.26 ± 2.25	85.69 ± 8.29	81.13 ± 3.48	87.36 ± 2.33*	84.48 ± 4.35	90.61 ± 3.16	112.45 ± 5.82	108.67 ± 11.19
100	93.77 ± 9.12	76.39 ± 3.77	92.38 ± 8.27	86.62 ± 2.49	78.62 ± 1.95	75.84 ± 4.93*	80.76 ± 6.71	100.74 ± 2.09*	107.9 ± 3.48	99.16 ± 4.41	94.98 ± 2.09*	97.16 ± 10.84

Values are presented as the mean % ± SD (standard deviation) of three independent experiments of cells viability compared to the control cells (not treated with sprouts extracts). Significant differences between the cells treated with the extracts from the Brassica sprouts grown in microgravity and/or darkness conditions versus control sprouts (for each plant, harvest day and concentration separately) were marked with an upper asterisk* ($p \leq 0.05$). Abbreviations of the sprouts' samples: B – broccoli; L – standard light (control); D – darkness; LM – standard light and simulated microgravity; DM – darkness and simulated microgravity. The numbers placed before each abbreviation indicate harvest days (5, 6, and 7 days).

Table S2. Viability of thyroid cancer (FTC-133, 8505C and TPC-1) and thyroid normal (Nthy-ori 3-1) cells after 24 h incubation with kale sprouts' extract.

[μg/mL]	5KaL	6KaL	7KaL	5KaLM	6KaLM	7KaLM	5KaD	6KaD	7KaD	5KaDM	6KaDM	7KaDM
						FTC-133						
50	97.75 ± 8.11	106.26 ± 6.89	94.74 ± 3.35	91.23 ± 3.08	111.47 ± 11.21	131.60 ± 8.84*	70.7 ± 7.21*	92.14 ± 3.78	112.97 ± 7.21	119.38 ± 5.77*	100.05 ± 10.09	109.76 ± 5.3
100	67.50 ± 4.59	92.14 ± 9.89	100.7 ± 5.48	104.16 ± 4.75*	95.09 ± 10.62	118.47 ± 11.73	76.71 ± 4.08	84.13 ± 2.56	91.34 ± 5.73	93.69 ± 4.46*	100.65 ± 4.2	82.92 ± 10.21
						8505C						
50	91.32 ± 5.01	83.68 ± 3.0	84.71 ± 7.5	95.59 ± 10.25	100.45 ± 2.73	95.65 ± 5.78	104.35 ± 1.55	93.42 ± 5.38	95.82 ± 3.16	102.36 ± 8.7	88.61 ± 13.84	114.37 ± 5.83*
100	116.76 ± 12.77	93.09 ± 2.65	99.79 ± 3.73	99.56 ± 12.32	116.35 ± 6.99*	96.89 ± 1.05	118.63 ± 5.09	87.78 ± 4.04	100.45 ± 5.46	100.21 ± 6.95	93.74 ± 5.36	104.18 ± 2.65
						TPC-1						

50	73.03 ± 5.06	69.42 ± 2.59	78.13 ± 2.24	90.25 ± 5.08*	90.44 ± 4.17*	82.26 ± 8.59	90.34 ± 3.76*	92.51 ± 4.82*	80.19 ± 5.06	85.73 ± 3.9	82.6 ± 7.1	97.94 ± 2.29*
100	75.34 ± 1.95	69.80 ± 3.84	76.92 ± 2.78	90.82 ± 10.58	74.81 ± 1.95	74.04 ± 1.64	73.75 ± 7.42	77.65 ± 7.73	78.03 ± 3.54	80.96 ± 3.74	75.77 ± 2.17	79.52 ± 10.63
							Nthy-ori 3-1					
50	114.10 ± 6.41	98.33 ± 1.53	92.67 ± 2.54	88.56 ± 4.82*	75.85 ± 4.17*	77.01 ± 4.04	83.26 ± 2.19*	83.04 ± 6.04	96.34 ± 4.92	86.08 ± 8.32*	77.96 ± 4.52*	107.88 ± 9.1
100	95.31 ± 4.79	98.72 ± 7.25	87.90 ± 3.09	97.77 ± 10.99	77.45 ± 2.09	87.10 ± 3.66	100.82 ± 3.39	100.82 ± 10.96	105.18 ± 11.62	101.33 ± 10.5	86.38 ± 1.85	111.26 ± 12.04*

Values are presented as the mean % ± SD (standard deviation) of three independent experiments of cells viability compared to the control cells (not treated with sprouts extracts). Significant differences between the cells treated with the extracts from the Brassica sprouts grown in microgravity and/or darkness conditions versus control sprouts (for each plant, harvest day and concentration separately) were marked with an upper asterisk* ($p \leq 0.05$). Abbreviations of the sprouts' samples: Ka – kale; L – standard light (control); D – darkness; LM – standard light and simulated microgravity; DM – darkness and simulated microgravity. The numbers placed before each abbreviation indicate harvest days (5, 6, and 7 days).

Table S3. Viability of thyroid cancer (FTC-133, 8505C and TPC-1) and thyroid normal (Nthy-ori 3-1) cells after 24 h incubation with kohlrabi sprouts' extract.

[µg/mL]	5KoL	6KoL	7KoL	5KoLM	6KoLM	7KoLM	5KoD	6KoD	7KoD	5KoDM	6KoDM	7KoDM
							FTC-133					
50	90.97 ± 10.5	104.58 ± 7.0	92.52 ± 3.54	76.88 ± 3.04	63.75 ± 5.83*	83.57 ± 11.9	104.5 ± 10.19	124.03 ± 18.08	111.02 ± 11.87	96.3 ± 12.79	106.96 ± 8.44	106.01 ± 8.5
100	95.42 ± 7.42	93.23 ± 6.95	97.49 ± 16.99	75.76 ± 10.29	71.87 ± 2.99	97.13 ± 6.75	117.79 ± 13.68	66.45 ± 6.16	121.05 ± 7.25	97.17 ± 12.59	103.26 ± 7.60	89.65 ± 12.66
							8505C					
50	105.28 ± 2.82	94.98 ± 3.79	93.11 ± 7.32	85.75 ± 1.41*	103.3 ± 6.03	96.83 ± 4.37	106.91 ± 8.81	101.64 ± 2.25	76.79 ± 1.55	79.02 ± 8.45*	93.76 ± 6.2	81.59 ± 3.47
100	120.13 ± 1.01	110.1 ± 7.42	110.37 ± 8.46	100.33 ± 4.88	104.42 ± 10.28	84.88 ± 2.99*	101.37 ± 3.96	113.98 ± 10.76	94.19 ± 3.75	91.02 ± 3.34*	95.90 ± 7.1	82.87 ± 12.83*
							TPC-1					
50	77.25 ± 1.06	77.98 ± 2.34	88.78 ± 4.43	84.54 ± 2.66	85.10 ± 9.79	72.36 ± 8.87	90.06 ± 7.12	89.89 ± 5.18	97.40 ± 2.53	88.55 ± 3.18	89.44 ± 16.86	88.83 ± 1.46
100	84.10 ± 7.64	88.22 ± 7.41	71.97 ± 4.67	78.76 ± 13.43	81.98 ± 5.93	76.81 ± 6.44	77.19 ± 4.60	80.98 ± 3.93	87.16 ± 3.84	77.98 ± 4.02	81.15 ± 8.36	75.64 ± 3.45
							Nthy-ori 3-1					
50	55.46 ± 2.36	99.17 ± 7.83	114.62 ± 4.26	99.0 ± 5.56*	99.41 ± 3.45*	112.55 ± 12.19	74.68 ± 7.29	77.42 ± 3.56	113.19 ± 2.33	108.43 ± 8.41*	83.23 ± 11.07	66.52 ± 4.76*
100	97.42 ± 5.66	76.32 ± 13.56	78.28 ± 11.11	100.66 ± 14.09	78.52 ± 4.22	66.52 ± 2.73	78.6 ± 7.47	89.03 ± 5.40	82.99 ± 6.25	86.13 ± 7.98	61.81 ± 8.54	62.52 ± 4.05

Values are presented as the mean % ± SD (standard deviation) of three independent experiments of cells viability compared to the control cells (not treated with sprouts extracts). Significant differences between the cells treated with the extracts from the Brassica sprouts grown in microgravity and/or darkness conditions versus control sprouts (for each plant, harvest day and concentration separately) were marked with an upper asterisk* ($p \leq 0.05$). Abbreviations of the sprouts' samples: Ko – kohlrabi; L – standard light (control); D – darkness; LM – standard light and simulated microgravity; DM – darkness and simulated microgravity. The numbers placed before each abbreviation indicate harvest days (5, 6, and 7 days).

Table S4. Viability of thyroid cancer (FTC-133, 8505C and TPC-1) and thyroid normal (Nthy-ori 3-1) cells after 24 h incubation with Brussels sprouts' extract.

[μg/mL]	5BSL	6BSL	7BSL	5BSLM	6BSLM	7BSLM	5BSD	6BSD	7BSD	5BSDM	6BSDM	7BSDM
						FTC-133						
50	83.85 ± 2.53	82.11 ± 3.41	77.08 ± 13.33	85.77 ± 4.57	60.44 ± 2.93*	58.79 ± 1.38*	57.69 ± 2.33*	54.13 ± 3.01*	54.95 ± 5.49*	59.71 ± 3.54*	73.51 ± 6.82	74.47 ± 1.36
100	87.50 ± 1.26	82.29 ± 1.64	85.04 ± 3.63	82.66 ± 3.54	52.03 ± 5.78*	54.04 ± 4.09*	63.18 ± 5.82*	55.96 ± 3.71*	76.17 ± 14.21	68.21 ± 8.81*	65.93 ± 2.23	77.22 ± 4.85
						8505C						
50	92.48 ± 1.71	80.26 ± 10.49	76.64 ± 4.33	82.52 ± 4.7	92.33 ± 7.99	85.99 ± 4.54	94.06 ± 6.4	85.84 ± 16.82	100.93 ± 5.49*	98.59 ± 2.09	109.32 ± 7.89*	107.99 ± 6.21*
100	105.38 ± 3.61	92.26 ± 7.55	85.01 ± 4.49	82.68 ± 3.99*	89.01 ± 10.62	96.78 ± 2.1	98.74 ± 6.03	120.05 ± 5.92*	112.52 ± 10.79*	101.38 ± 13.88	109.43 ± 7.26	121.6 ± 7.09*
						TPC-1						
50	102.30 ± 3.75	103.05 ± 8.86	110.56 ± 6.3	99.22 ± 11.48	135.11 ± 6.16*	99.64 ± 5.28	111.36 ± 3.6	99.55 ± 15.58	124.61 ± 16.39	114.58 ± 4.13	101.32 ± 10.74	117.66 ± 4.02
100	119.38 ± 5.96	106.13 ± 10.33	114.25 ± 8.61	106.46 ± 6.48	99.36 ± 3.25	80.13 ± 6.64*	113.69 ± 5.77	112.52 ± 12.23	118.45 ± 5.9	117.66 ± 4.97	97.26 ± 10.53	82.53 ± 2.48*
						Nthy-ori 3-1						
50	95.34 ± 2.41	81.52 ± 5.32	89.29 ± 4.95	89.45 ± 2.81	72.33 ± 7.66	112.93 ± 5.56*	97.3 ± 5.11	94.56 ± 0.76	86.86 ± 9.22	85.05 ± 6.25	85.29 ± 7.66	101.66 ± 2.89
100	92.98 ± 6.87	73.04 ± 8.89	96.36 ± 2.33	91.65 ± 4.63	77.98 ± 7.96	113.19 ± 8.38	109.32 ± 8.57	109.48 ± 8.61*	116.31 ± 6.21	89.45 ± 4.68	89.53 ± 8.21	114.66 ± 1.21

Values are presented as the mean % ± SD (standard deviation) of three independent experiments of cells viability compared to the control cells (not treated with sprouts extracts). Significant differences between the cells treated with the extracts from the Brassica sprouts grown in microgravity and/or darkness conditions versus control sprouts (for each plant, harvest day and concentration separately) were marked with an upper asterisk* ($p \le 0.05$). Abbreviations of the sprouts' samples: BS – Brussels sprouts; L – standard light (control); D – darkness; LM – standard light and simulated microgravity; DM – darkness and simulated microgravity. The numbers placed before each abbreviation indicate harvest days (5, 6, and 7 days).

Table S5. The antiproliferative activity of broccoli sprouts (50 μg/mL).

	5BL	6BL	7BL	5BLM	6BLM	7BLM	5BD	6BD	7BD	5BDM	6BDM	7BDM
						FTC-133						
24h	113.06 ± 12.60	93.09 ± 4.06	76.30 ± 7.29	89.30 ± 9.77*	91.86 ± 4.59	86.84 ± 9.18	86.23 ± 4.65*	86.33 ± 6.67	86.23 ± 10.59	81.62 ± 2.46*	89.61 ± 8.01	90.73 ± 4.32
48h	80.46 ± 2.51	76.64 ± 5.08	67.67 ± 2.70	86.11 ± 2.96	97.54 ± 6.15*	93.29 ± 10.85*	74.60 ± 9.47	72.94 ± 3.54	73.79 ± 7.80	67.08 ± 1.98	81.39 ± 4.19	86.83 ± 5.04*
72h	80.22 ± 9.26	44.23 ± 2.88	85.75 ± 3.55	89.35 ± 4.17	88.60 ± 7.63*	72.52 ± 4.24	76.19 ± 13.80	83.70 ± 4.58*	76.46 ± 1.72	63.77 ± 5.14	80.39 ± 11.2*	60.48 ± 4.27*
						8505C						
24h	87.61 ± 2.25	88.41 ± 0.68	86.53 ± 0.98	97.16 ± 7.64	96.62 ± 3.48	100.70 ± 6.31	92.54 ± 8.43	93.40 ± 8.13	95.54 ± 6.09	99.41 ± 9.24	107.78 ± 3.47*	111.76 ± 11.40*
48h	97.49 ± 8.08	109.22 ± 2.29	98.98 ± 5.34	98.50 ± 6.01	100.73 ± 12.90	100.12 ± 3.15	90.21 ± 5.88	92.77 ± 7.07	93.99 ± 4.69	87.92 ± 3.37	89.94 ± 4.79	104.10 ± 9.71
72h	83.91 ± 5.28	88.20 ± 8.07	76.65 ± 5.92	84.67 ± 4.31	82.58 ± 3.47	80.50 ± 2.02	77.77 ± 2.78	76.20 ± 3.92	76.32 ± 2.06	80.26 ± 4.7	79.94 ± 5.18	87.40 ± 6.89
						TPC-1						

24h	95.54 ± 10.12	87.55 ± 2.01	94.34 ± 1.08	99.49 ± 7.93	100.66 ± 5.18	105.30 ± 11.60	88.31 ± 1.86	102.23 ± 8.52	108.80 ± 8.52	114.68 ± 7.89*	110.88 ± 3.11*	117.60 ± 4.31*
48h	77.82 ± 5.56	74.57 ± 4.19	73.65 ± 5.69	81.03 ± 3.35	83.96 ± 1.62	81.31 ± 3.38	75.94 ± 6.19	68.42 ± 3.27	68.84 ± 5.03	83.75 ± 6.80	95.58 ± 3.30*	91.88 ± 11.40*
72h	75.01 ± 4.21	68.99 ± 8.05	66.78 ± 5.56	69.31 ±7.07	55.36 ± 0.38	59.42 ± 41	54.99 ± 6.22*	53.17 ± 2.87*	65.55 ± 1.52	56.88 ± 3.55*	58.57 ± 4.20	59.07 ± 2.11
Nthy-ori 3-1												
24h	71.39 ± 11.50	62.43 ± 5.84	67.51 ± 8.46	73.68 ± 3.59	68.36 ± 2.00	62.07 ± 1.03	57.89 ± 6.93	56.02 ± 2.06	53.00 ± 5.70	67.63 ± 8.04	74.77 ± 4.11	62.31 ± 5.09
48h	60.64 ± 5.46	44.80 ± 4.23	50.63 ± 5.39	54.23 ± 4.58	55.20 ± 9.42	44.31 ± 2.31	40.14 ± 5.72*	38.00 ± 1.68	37.12 ± 3.88	41.40 ± 5.68*	46.06 ± 5.74	46.16 ± 4.81
72h	50.71 ± 11.10	55.54 ± 8.95	44.17 ± 1.52	45.89 ± 8.71	45.25 ± 9.05	43.10 ± 4.95	40.03 ± 3.42	37.96 ± 0*	38.88 ± 3.12	38.74 ± 3.12	37.67 ± 4.18*	40.53 ± 4.52

The results are presented as the mean % of cells proliferation inhibition ± SD (standard deviation) of three independent experiments. Significant differences between the cells treated with the extracts from the Brassica sprouts grown in microgravity and/or darkness conditions versus control sprouts were marked with an upper asterisk* ($p \leq 0.05$). Abbreviations of the sprouts' samples: B – broccoli; L – standard light (control); D – darkness; LM – standard light and simulated microgravity; DM – darkness and simulated microgravity. The numbers placed before each abbreviation indicate harvest days (5, 6, and 7 days).

Table S6. The antiproliferative activity of kale sprouts (50 μg/mL).

	5KaL	6KaL	7KaL	5KaLM	6KaLM	7KaLM	5KaD	6KaD	7KaD	5KaDM	6KaDM	7KaDM
							FTC-133					
24h	98.41 ± 13.10	86.74 ± 7.86	82.03 ± 5.69	72.50 ± 4.26*	70.76 ± 3.67	74.04 ± 5.85	91.09 ± 1.96	94.32 ± 3.48	79.06 ± 8.04	89.09 ± 10.40	94.47 ± 6.30	92.06 ± 7.84
48h	69.46 ± 6.60	53.02 ± 2.76	46.60 ± 8.87	57.39 ± 3.39	51.74 ± 7.73	52.59 ± 4.59	49.06 ± 3.60*	42.31 ± 3.60	36.89 ± 0.99	53.65 ± 6.33	43.84 ± 2.34	63.25 ± 13.45
72h	62.39 ± 6.16	57.73 ± 14.29	39.21 ± 1.34	64.28 ± 2.73	49.80 ± 4.69	42.61 ± 10.58	73.38 ± 4.16	45.23 ± 2.72	34.35 ± 2.90	44.13 ± 2.06	47.60 ± 13.50	51.85 ± 4.79
							8505C					
24h	117.23 ± 7.74	106.12 ± 15.30	105.96 ± 6.54	99.84 ± 8.13	104.45 ± 8.54	107.46 ± 3.82	104.35 ± 2.01	100.16 ± 5.92	103.81 ± 3.18	109.50 ± 2.73	106.76 ± 16.60	98.12 ± 4.09
48h	109.02 ± 6.58	102.75 ± 0.57	99.65 ± 5.52	98.70 ± 6.36	101.13 ± 4.92	108.96 ± 2.23	89.00 ± 6.29*	101.00 ± 8.42	93.99 ± 7.35	87.78 ± 3.46*	93.58 ± 7.13	87.78 ± 9.73
72h	106.74 ± 6.88	108.83 ± 8.88	94.06 ± 2.97	104.33 ± 4.24	94.86 ± 5.79	88.84 ± 5.66	93.42 ± 5.88	91.25 ± 8.18	93.18 ± 9.22	91.81 ± 8.34	99.20 ± 7.69	102.09 ± 6.68
							TPC-1					
24h	99.82 ± 3.80	122.70 ± 12.50	91.62 ± 11.81	89.55 ± 9.72	86.13 ± 5.49*	67.75 ± 6.75*	95.81 ± 7.07	102.43 ± 1.15	105.86 ± 11.00	103.11 ± 2.48	101.89 ± 7.04	93.11 ± 11.70
48h	69.20 ± 10.74	77.18 ± 0.22	65.45 ± 7.56	67.86 ± 4.94	66.44 ± 9.57	29.071 ± 3.83*	78.38 ± 6.97	74.25 ± 6.13	65.45 ± 0.96	78.88 ± 3.38	84.03 ± 2.55	78.89 ± 5.47
72h	75.51 ± 4.71	73.87 ± 5.38	74.04 ± 9.71	76.09 ± 1.84	71.83 ± 5.27	26.71 ± 4.69*	80.00 ± 5.18	65.91 ± 4.69	60.49 ± 2.51	68.69 ± 6.52	62.67 ± 6.84	66.64 ± 1.59
							Nthy-ori 3-1					
24h	76.35 ± 6.74	61.34 ± 4.40	52.63 ± 2.51	49.61 ± 7.80*	49.91 ± 3.85	52.15 ± 3.29	57.96 ± 5.10*	64.97 ± 6.67	53.24 ± 1052	53.90 ± 5.39*	52.27 ± 4.99	53.36 ± 0.96
48h	49.37 ± 9.06	49.56 ± 0.41	52.38 ± 1.98	48.69 ± 6.39	45.67 ± 5.46	44.61 ± 5.62	39.50 ± 2.27	49.08 ± 4.63	44.90 ± 3.31	46.16 ± 0.84	49.66 ± 4.42	47.52 ± 11.50
72h	43.82 ± 1.31	36.17 ± 2.68	32.09 ± 0.89	31.02 ± 2.04*	30.02 ± 1.50	31.24 ± 1.46	34.24 ± 6.77	36.88 ± 6.15	36.17 ± 3.28	33.38 ± 4.24	34.60 ± 2.15	33.95 ± 4.03

The results are presented as the mean % of cells proliferation inhibition ± SD (standard deviation) of three independent experiments. Significant differences between the cells treated with the extracts from the Brassica sprouts grown in microgravity and/or darkness conditions versus control sprouts were marked with an upper asterisk* ($p \le 0.05$). Abbreviations of the sprouts' samples: Ka – kale; L – standard light (control); D – darkness; LM – standard light and simulated microgravity; DM – darkness and simulated microgravity. The numbers placed before each abbreviation indicate harvest days (5, 6, and 7 days).

Table S7. The antiproliferative activity of kohlrabi sprouts (50 μg/mL).

	5KoL	6KoL	7KoL	5KoLM	6KoLM	7KoLM	5KoD	6KoD	7KoD	5KoDM	6KoDM	7KoDM
							FTC-133					
24h	128.31 ± 7.31	115.51 ± 9.76	107.07 ± 2.39	87.40 ± 5.00*	97.80 ± 2.90	78.65 ± 6.95*	105.68 ± 4.34*	93.24 ± 11.50*	88.17 ± 10.43	93.71 ± 3.91*	92.88 ± 10.20*	88.79 ± 9.23
48h	93.29 ± 5.19	81.90 ± 3.82	73.79 ± 14.15	79.72 ± 12.70	80.29 ± 0.36	70.90 ± 4.91	69.41 ± 14.20	62.62 ± 9.87	66.53 ± 10.72	71.45 ± 10.90	88.25 ± 1.17	94.99 ± 4.84
72h	87.56 ± 4.13	80.05 ± 6.03	77.93 ± 5.66	73.07 ± 9.75	82.02 ± 7.00	72.66 ± 3.71	80.80 ± 6.27	80.91 ± 6.07	78.09 ± 3.82	67.87 ± 5.63*	75.85 ± 5.54	82.90 ± 10.65
							8505C					
24h	108.96 ± 3.90	102.31 ± 2.90	95.65 ± 6.34	97.15 ± 4.08	103.70 ± 3.07	108.53 ± 8.83	100.70 ± 6.44	106.71 ± 11.70	101.02 ± 7.77	89.53 ± 0.91	104.24 ± 10.40	90.02 ± 12.50

48h	143.81 ± 7.72	118.12 ± 5.72	111.25 ± 10.30	109.76 ± 4.86*	112.26 ± 1.43	116.64 ± 5.52	105.31 ± 8.29*	101.94 ± 0.57	103.69 ± 3.99	103.56 ± 5.66*	104.57 ± 2.00	110.17 ± 5.39
72h	112.68 ± 0.34	111.56 ± 7.79	108.95 ± 2.89	99.52 ± 5.18	98.55 ± 8.26	103.29 ± 2.44	102.81 ± 14.50	103.13 ± 6.15	100.52 ± 14.50	103.85 ± 3.75	112.92 ± 8.45	108.19 ± 5.37
TPC-1												
24h	133.96 ± 6.78	107.12 ± 9.41	119.32 ± 4.78	88.92 ± 5.95*	133.65 ± 4.40*	130.68 ± 6.69	115.41 ± 7.61*	112.16 ± 9.17	116.67 ± 5.58	116.49 ± 1.89	114.41 ± 3.07	98.29 ± 10.06*
48h	115.65 ± 5.87	66.34 ± 4.05	103.04 ± 6.38	45.64 ± 4.61*	104.38 ± 9.74*	104.17 ± 8.24	104.24 ± 9.54	100.49 ± 8.94*	101.73 ± 6.98	95.73 ± 6.06*	105.05 ± 4.19*	97.44 ± 12.96
72h	87.57 ± 4.72	73.80 ± 3.35	78.06 ± 5.47	52.85 ± 5.70*	73.49 ± 3.17	67.05 ± 7.96	68.45 ± 7.03	64.78 ± 4.26	74.27 ± 9.14	79.03 ± 8.96	77.60 ± 4.73	85.22 ± 11.86
Nthy-ori 3-1												
24h	96.37 ± 1.80	81.79 ± 8.37	66.97 ± 5.90	66.30 ± 4.33*	75.14 ± 0.51	58.20 ± 4.93	75.32 ± 4.36*	79.13 ± 7.50	75.26 ± 7.53	65.34 ± 2.62*	66.42 ± 6.67	72.84 ± 8.26
48h	65.89 ± 6.31	54.62 ± 4.46	61.90 ± 0.73	55.88 ± 9.34	60.45 ± 4.09	48.59 ± 3.56	45.87 ± 2.55*	65.11 ± 11.30	64.82 ± 3.69	50.73 ± 2.81	57.24 ± 5.07	75.36 ± 3.50
72h	66.55 ± 9.43	39.99 ± 8.95	39.56 ± 1.67	35.10 ± 0.97*	39.56 ± 7.13	35.03 ± 4.03	41.89 ± 8.36*	59.69 ± 7.95*	60.36 ± 4.70*	33.95 ± 3.54*	37.03 ± 3.98	54.79 ± 3.49

The results are presented as the mean % of cells proliferation inhibition ± SD (standard deviation) of three independent experiments. Significant differences between the cells treated with the extracts from the Brassica sprouts grown in microgravity and/or darkness conditions versus control sprouts were marked with an upper asterisk* ($p \leq 0.05$). Abbreviations of the sprouts' samples: Ko – kohlrabi; L – standard light (control); D – darkness; LM – standard light and simulated microgravity; DM – darkness and simulated microgravity. The numbers placed before each abbreviation indicate harvest days (5, 6, and 7 days).

Table S8. The antiproliferative activity of Brussels sprouts' sprouts (50 μg/mL).

	5BSL	6BSL	7BSL	5BSLM	6BSLM	7BSLM	5BSD	6BSD	7BSD	5BSDM	6BSDM	7BSDM
FTC-133												
24h	119.41 ± 22.90	106.00 ± 0.87	91.76 ± 14.21	103.64 ± 13.50	98.82 ± 5.51	92.99 ± 7.96	107.01 ± 3.29	108.35 ± 13.50	84.59 ± 11.46	90.22 ± 2.05	93.60 ± 4.48	98.82 ± 10.47
48h	92.01 ± 2.36	73.24 ± 5.76	67.59 ± 1.85	89.12 ± 10.80	72.77 ± 8.72	90.27 ± 1.36	85.77 ± 9.35	83.39 ± 6.63	67.76 ± 10.63	76.08 ± 2.52	75.74 ± 6.70	76.15 ± 0.81
72h	94.35 ± 6.58	54.72 ± 6.28	45.05 ± 3.89	93.62 ± 3.51	64.56 ± 15.70	83.36 ± 4.83*	80.66 ± 8.55	81.86 ± 2.19*	61.96 ± 16.11	75.49 ± 5.13	77.36 ± 9.49	73.04 ± 3.32*
8505C												
24h	110.57 ± 5.14	106.60 ± 1.82	94.20 ± 5.69	101.34 ± 7.73	105.10 ± 7.33	106.28 ± 11.20	96.83 ± 8.24	101.88 ± 8.05	96.40 ± 4.25	101.13 ± 7.87	106.12 ± 7.06	108.05 ± 13.40
48h	134.91 ± 2.00	103.76 ± 11.20	101.94 ± 4.00	110.71 ± 4.09*	104.37 ± 9.81	98.44 ± 5.67	97.09 ± 0.40*	90.21 ± 2.83	86.03 ± 1.53*	89.13 ± 4.69*	89.27 ± 8.17	93.18 ± 3.14
72h	87.56 ± 8.48	88.20 ± 3.48	79.78 ± 4.06	89.33 ± 5.90	90.45 ± 3.21	85.07 ± 5.78	86.04 ± 1.64	85.47 ± 4.61	80.42 ± 2.41	86.92 ± 2.30	86.76 ± 4.10	88.28 ± 6.81
TPC-1												
24h	116.80 ± 5.89	114.10 ± 9.37	101.90 ± 9.11	119.87 ± 8.68	92.91 ± 4.53*	105.92 ± 4.81	111.10 ± 6.17	130.17 ± 9.83	119.50 ± 10.07	114.83 ± 5.89	110.01 ± 6.47	111.76 ± 0.79
48h	82.97 ± 5.39	77.32 ± 11.41	64.61 ± 13.92	97.95 ± 7.44	69.69 ± 7.14	89.30 ± 6.37*	99.33 ± 5.07	106.32 ± 9.66*	93.68 ± 4.48*	115.82 ± 0.45*	96.54 ± 1.35	92.72 ± 7.34*
72h	68.03 ± 5.19	65.58 ± 2.50	59.57 ± 7.30	72.76 ± 9.95	55.40 ± 6.84	64.73 ± 9.04	62.76 ± 9.33	68.77 ± 4.53	60.63 ± 7.10	56.66 ± 3.67	62.32 ± 0.89	72.39 ± 0.90
Nthy-ori 3-1												

24h	71.39 ± 11.50	72.23 ± 11.00	78.58 ± 10.00	75.62 ± 8.26*	51.54 ± 0.51*	63.34 ± 5.39	82.27 ± 6.70*	73.00 ± 5.84	67.70 ± 2.82	70.78 ± 5.59*	76.23 ± 1.54	60.50 ± 6.12*
48h	103.89 ± 7.87	80.76 ± 13.60	55.30 ± 9.87	64.33 ± 11.10*	46.65 ± 2.06*	57.43 ± 8.49	68.08 ± 1.03*	65.31 ± 2.20	50.73 ± 9.34	67.20 ± 10.90*	64.04 ± 9.48	65.79 ± 6.71
72h	84.99 ± 11.60	59.19 ± 6.87	56.11 ± 11.70	68.08 ± 2.58	45.46 ± 0.61	44.60 ± 1.52	53.40 ± 2.43*	54.47 ± 0.61	51.82 ± 6.28	71.34 ± 9.36	44.89 ± 8.76	43.10 ± 2.73

The results are presented as the mean % of cells proliferation inhibition ± SD (standard deviation) of three independent experiments. Significant differences between the cells treated with the extracts from the Brassica sprouts grown in microgravity and/or darkness conditions versus control sprouts were marked with an upper asterisk* ($p \leq 0.05$). Abbreviations of the sprouts' samples: BS – Brussels sprouts; L – standard light (control); D – darkness; LM – standard light and simulated microgravity; DM – darkness and simulated microgravity. The numbers placed before each abbreviation indicate harvest days (5, 6, and 7 days).

Table S9. The details of PCA model for subset of all original response.

Component	Percentages of explained variation (R^2)	Percentage of total variation predicted by component (Q^2)	Eigenvalues
t1	30.2	20.4	9.68
t2	20.4	19.2	6.54
t3	9.2	4.7	2.94
t4	7.0	0.1	2.25

t1 was mainly loaded by: 24h FTC-133 CV50 (-0.25), 48h TPC-1 CV50 (-0.23), 24h Nthy-ori 3-1 CV50 (-0.26), 24h FTC-133 CV100 (-0.26), 48h FTC-133 CV100 (-0.24), 72h FTC-133 CV100 (-0.27); t3 was mainly loaded by: 8505C MTT 50 (0.30), 8505C MTT 300 (0.36), 72h TPC-1 CV100 (0.34)

Samples' abbreviations in chemometric analysis:

- 72h 8505C CV50 - antiproliferative assay, concentration of 50 µg/mL, 72h of incubation, 8505C cells;
- 24h 8505C CV100 - antiproliferative assay, concentration of 100 µg/mL, 24 h of incubation, 8505C cells;
- 72h 8505C CV100 - antiproliferative assay, concentration of 100 µg/mL, 72 h of incubation, 8505C cells;
- TPC-1 MTT 300 - cytotoxic assay, concentration of 300 µg/mL, TPC-1 cells;
- Nthy-ori 3-1 MTT 300 - cytotoxic assay, concentration of 300 µg/mL, Nthy-ori 3-1 cells;
- 72h FTC-133 CV50 - antiproliferative assay, concentration of 50 µg/mL, 72h of incubation, FTC-133 cells;
- 24h 8505C CV50 – antiproliferative assay, concentration of 50 µg/mL, 24 h of incubation, 8505C cells;
- FTC-133 MTT 300 - cytotoxic assay, concentration of 300 µg/mL, FTC-133 cells.
